# A Capacitated House Allocation Game for the Energy Efficient Relays Selection in 5G Multicast Context

**DOI:** 10.3390/s20185347

**Published:** 2020-09-18

**Authors:** Francesco Chiti, Romano Fantacci, Benedetta Picano, Laura Pierucci

**Affiliations:** Department of Information Engineering, Università di Firenze, Via di Santa Marta 3, 50139 Firenze, Italy; francesco.chiti@unifi.it (F.C.); romano.fantacci@unifi.it (R.F.); laura.pierucci@unifi.it (L.P.)

**Keywords:** relay selection, device-to-device communications, matching game, multicast context

## Abstract

The upcoming fifth generation (5G) wireless networks making use of higher-frequency spectrum bands suffer from serious propagation issues due to high path loss and beam directivity requirements. This promotes the device-to-device communications to boost the transmission reliability at the network edges, providing remarkable benefits in terms of the energy and spectrum efficiency, essential for a wide class of sensors networks and Internet-of-Things. More in general, applications where devices are usually constrained in computational and transmission range capabilities. In such a context, the selection of the proper number of devices arranged as a relay plays a crucial role. Towards this goal, this paper proposes an efficient relay selection scheme minimizing both the delivery transmission delay and the overall energy consumption, i.e., the overall number of relays to be used. By focusing on a multicast content delivery application scenario the problem of interest is formulated as a one-sided preferences matching game. In addition, the strategy designed takes into account specific information, named reputation coefficient, associated to each device jointly with link propagation conditions for allowing the selection of suitable relays for disseminating the content among the devices. The effectiveness of the proposed solution is underpinned by computer simulations, and the performance is evaluated in terms of power consumption, end-to-end delay, and number of selected relays. As confirmed by results, the proposed approach improves network performance compared to the greedy approach, the random algorithm, a scheme previously proposed in literature, and with two game theory-based strategies.

## 1. Introduction

The fifth generation (5G) cellular networks suffering from severe propagation issues massively promote the device-to-device (D2D) communication paradigm, i.e., direct connections between users, to improve the communications reliability at the network edges [1,2,3]. In the cooperative communications, already included in the previous fourth generation Long Term Evolution-Advanced cellular networks, fixed low-power nodes are arranged to support communication between the base station and the end-devices performing spatial diversity gains. As widely proved in the literature [1,4,5], the introduction of the devices acting as a relay may significantly improve the network performance. This emerging paradigm, known as indirect network connection mode, represents a killer feature of the upcoming 5G technology to counteract the negative effects of the channel propagation conditions at the network edges, by allowing D2D communications directly between devices if the line of sight (LOS) signals are not available in a reliable manner. The advantages deriving by the D2D communications are appreciable in terms of (i) improvements on offloading flexibility; (ii) reduction of the discovery procedures cost for the context-aware services and applications; (iii) improvements in resource utilization; (iv) latency and energy consumption reduction [6,7].

Therefore, in 5G environments, the proper relays selection plays an essential role for designing networks able of energy efficient short-range D2D communications [8,9].

The paper investigates the problem of multicast communications considering relay-based schemes operating in the decoding-and-foreword mode and D2D communications to share a same content simultaneously from a source to a target users community according to the multicast paradigm. In particular, we focus here on a relays selection policy based on the the joint use of devices reputation information [10,11] and link propagation conditions.

Generally speaking, the reputation coefficient may assume a wide range of meanings, from the hardware capabilities such as the battery life time, to the security level, to name a few. Therefore, with the aim at proposing a general purpose relay selection framework, we have left unspecified the meaning of the reputation coefficient in what follows.

This paper focuses on optimizing the content delivery delay (defined as the minimum time period in which all the devices retrieve successfully the interesting content) and the number of the involved relays with the aim at minimizing the overall energy consumption, by adopting a matching theory (MT) approach and supposing one shared channel. Therefore, the main contributions of this paper are summarized as follows

- We propose a relay-based content delivery approach to offload the data traffic through D2D communications, by establishing energy efficient connections. In particular, multiple relays can be selected among all the interested users to spread the content and minimize the end-to-end delivery delay.
- The adopted matching process achieves the combinations of relays and destinations according to a reputation strategy and the propagation conditions of the links from source to relays and the links from relays to end-users with respect to the direct link (i.e., from source to end-users). This joint process minimizes the worst delivery delay and the number of the involved relays, by resorting to the capacitated house allocation matching problem.

The rest of paper is organized as follows. In Section 2 and in-depth literature review is provided. In Section 3, the system model is detailed and the proposed strategy is presented in Section 4. In Section 5, the experimental results are shown. Finally, the conclusions are drawn in Section 6.

## 2. Related Works

Recently, several efforts have been made in providing schemes to efficiently perform D2D communications, especially in 5G networks. Some examples are represented by papers [1,5,12], that detail the challenges of the D2D communications contextualized to the 5G cellular networks. The problem of the relay selection for the point-to-point communications in the absence of the LOS has been investigated in [13,14,15].

The improvements in outage probability and capacity deriving by the introduction of the nodes acting as relays are analyzed in paper [16], showing the close forms for the ergodic multicast capacity and the outage probability. The optimal joint problem of the relay selection and the channel assignment is investigated in paper [17], by using a lexicographic max-min approach. The relay selection issue has been addressed also in paper [18], in which a distributed scheme is realized on the basis of a maximum distance threshold, involving the exchange of local tables to select the suitable relays. Roughly speaking, these relay selection policies choose the best propagation link analyzing the instantaneous channel state information in a network assisted vision.

A game theory-based approach has been shown in paper [19], in which a Stackelberg game has been formulated in order to optimize both the interference management and the D2D offloading, taking into account also the monetary aspect. Differently, the social behavior of users, which frequently use on-line social applications, such as Facebook, Twitter, YouTube, Instagram, and so on, has suggested the establishment of D2D groups in which each participant has the same interest in content sharing to drive cooperative D2D/relaying communications design [20,21]. The maximization of the amount of data relayed through D2D communications and the stability of the D2D transmissions are the main target of paper [22] for social networks. Similarly, the communications stability is taken into account even in [23], where large scale machine-to-machine (M2M) networks are well investigated. The authors in [23] pose emphasis on the fact that the social awareness during relay selection positively impacts on the network performance, providing improvements also in reference to the stability of communications. The problem of the throughput maximization of the network is studied in paper [24]. In this case, the relay selection scheme considers both the distance between the relay and the source–destination pair, and the corresponding social trust coefficient. The discovery procedure and the resource allocation problem contextualized to the content delivery issue is studied in [25], showing a two-sided preferences matching approach, considering both the social connections and the physical information, aiming at maximizing the sum rate for the D2D communities. A matching approach has been adopted also in paper [26], where the main objective was the maximization of the energy efficiency by keeping high levels of quality of service. Similarly to the work [25], our paper considers the same problem, i.e., the content delivery to end-users through relays. Differently, we focus our analysis on the of end-to-end delivery delay problem for multicast communications. Therefore, a relay selection is proposed, where each device can act as a relay by resorting to a one-sided preferences matching game, formulated as a capacitated house allocation matching problem [27]. Despite several papers proposing the matching theory as a solution as in [28], the problem of multicast delivery has not been taken into account.

## 3. System Model

### 3.1. Reference Scenario

**Figure 1:**
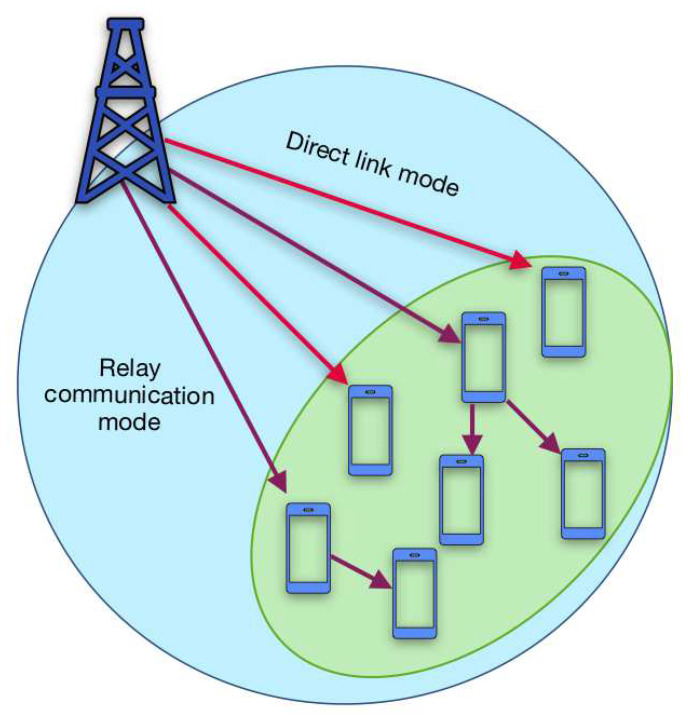
The reference network consists of a source node S and of a set of end devices (EDs) D.

As depicted in Figure 1, this paper considers as reference scenario a D2D network composed by one source node (SN) S, usually the eNodeB, and by a set of end devices (EDs) D={1,…,n}. It is important to highlight that the proposed framework may be easily contextualized to a sensors network scenario, in which the information has to be spread from a source S towards a sensors community D. The SN S, located far away from the set of EDs D, has to transmit the same information of size *L* in bits towards the whole ED community D, sited at the network edges. The ED elements of D are geographically close to each other and $G_{d}=\left(D_{d},E_{d}\right)$ denotes the EDs’ proximity graph, in which the vertices set $D_{d}$ is the ED set D, i.e., $D_{d}=D$, and $E_{d}$ is the edges set.

Furthermore, two EDs are connected through one edge if their mutual distance is such as to allow the D2D one hop communication in a reliable mode. $G_{d}$ is an undirected graph with a reputation coefficient $\rho_{i}$, i∈{1,…,n} associated to each node $i\in D_{d}$. A reputation coefficient $\rho_{i}\in \left(0,1\right)$ is relative to every single node *i* and expresses the reputation of the vertices of $G_{d}$. The degree of each vertex *i* of $G_{d}$, i.e., the number of edges incident on *i*, is denoted with $deg^{d}\left(i\right)$.

The transmission delay of packet *l* from the SN S to the ED *i* depends on whether *l* is sent through the direct link between S an *i* or with the one hop communication mode, selecting a node *j*, j≠i, as relay. We consider for the channel model, the path loss model for large-scale fading and a block fading Raleigh channel with the channel coefficient that consequently remains unchanged only during the transmission of one packet. Hence, the transmission delay of the direct link between S and *i* can be expressed as

(1) $$\delta_{S,i}=\frac{L}{\log_{2}\left(1+\zeta_{S,i}\right)}$$

where $\zeta_{S,i}$ represents the mean signal-to-noise-ratio (SNR) between S and *i*, measured at the ED *i* site. The mutual interference at the relays devices is not considered here by assuming the use of a dedicated transmission channel and ideal orthogonal transmissions on the other links. Furthermore, we assumed the use of a same shared channel to arrange communications between the selected relays and the EDs unable to have a reliable direct connection with the eNodeB. No perfect synchronization for the second hop transmissions, i.e., from the selected relays to the associated EDs, is considered. Consequently, the impairments due to the mutual interference effects at each ED connected to the appropriate relay due to the interfering transmissions of all the other relays has to be taken into account. Hence, for each ED *i* linked to the relay *j*, we have

(2) $$\delta_{S,j,i}=\delta_{S,j}+\frac{L}{\log_{2}\left(1+\zeta_{i,j}\right)},$$

where $\zeta_{i,j}$ is the mean signal-to-inteference-plus-noise ratio (SINR) at each device ED *i* reached by the relay *j*.

Hence, for each ED *i*, we have that the overall delay transmission from the SN S can be defined as

(3) $$\Delta_{i}=x_{i}\delta_{S,i}+\left(1-x_{i}\right)\sum_{j\in V_{i}}\delta_{S,j,i}\mu_{j,i},$$

where $x_{i}$ is a binary variable that assumes value 1 if the SN S packet *l* to ED *i* using the direct mode, 0 otherwise. Then, $V_{i}$ expresses the set of the EDs connected to *i* in the graph $G_{d}$, i.e., the set of all the possible relays for ED *i*. Finally, $\mu_{j,i}$ is 1 when ED *j* acts as relay for *i*, 0 otherwise. Whatever the transmission mode was, the whole D2D community has to receive *l*, and each ED *i* receives the packet *l* through only one link, given by

(4) $$x_{i}+\sum_{j\in V_{i}}\mu_{j,i}=1,\;\forall i\in D$$

with

(5) $$\sum_{j\in V_{i}}\mu_{j,i}\leq 1,\;\forall i\in D$$

(6) $$x_{i},\mu_{j,i}\in \left\{0,1\right\},\;\forall i,j\in \left\{1,\ldots ,n\right\}$$

### 3.2. Problem Formulation

This paper addresses the relays selection problem (RSP), minimizing both the delivery transmission delay and the number of the involved relays, in order to send the packet *l* to the whole D2D community. In formal terms, the RSP can be expressed as follows

(7) $$\min\max_{i\in D}\Delta_{i}\;and\;\min R$$

where R is the number of the selected relays in the considered D2D community. The problem represented by (4)–(7) aims at minimizing the delivery transmission delay and the number of the selected relays. Therefore, we propose an MT [29] based strategy to select the relays. Despite there not existing any theoretical result that strictly connects the optimal solution of problem (7)–(6) with the solution achieved by the application of MT, it is important to highlight that MT represents a powerful and low-complexity mathematical framework to reach a suboptimal solution of (7)–(6) which, as the number of EDs increases, exhibits a remarkable complexity.

## 4. Proposed Solution and Algorithm

Recently, MT has gained attention providing powerful tools to mathematically model the mutual satisfaction of elements belonging to two distinct sets, in being matched together. Specifically, MT optimally matches the elements of two sets taking into account the preferences expressed by each of these towards the elements of the opposite set and vice versa. MT is suitable to reach distributed optimal solutions, but still performs well in centralized scenarios. MT is widely used in many application areas, especially in wireless resource allocation problems as in [29,30,31]. One of the most known matching problems is the capacitated house allocation problem (CHA) [27,32], in which there is one set of residents and one set of houses, each of which can potentially be assigned to more than one resident until some fixed capacity *r*. In the CHA problem, the houses do not have preference lists over residents.

### 4.1. The RSP as an Instance of the CHA Problem

In our problem, the set of residents coincides with the ED set D, and the number of houses is equal to the number of possible communication modes, in our case two sets $S^{\prime }$ and $S^{\prime \prime }$. More in depth, $S^{\prime }$ and $S^{\prime \prime }$ represent the SN S distinguishing between the direct link and the relay communication mode, respectively. One crucial difference between the CHA problem and the RSP, is that in the CHA each house has a maximum number of residents that can accept, while in the RSP *r*, i.e., the number of selected relays is dynamic and changes based on the $G_{d}$ topology that changes, as detailed later, during the relay selection process. In particular, as introduced in Section 3 with the constraint (4), at the end of the proposed strategy each ED in D has received the packet *l* from one and only one sender (SN S or a nearby ED). In order to guarantee such an objective, how many and which relays have to be selected to cover all the vertices both depend on the connectivity of $G_{d}$. The proposed matching game acts based on the ED D preference lists that are built as follows.

### 4.2. EDs Preference List

Let $deg^{d}\left(i\right)$ be the degree of vertex *i* in $G_{d}$, hence the number of incident edges on *i* in $G_{d}$. The preference list is built considering, for each *i* in D, the maximum delay ${\bar{\delta }}_{i,j}$ associated to its incident edges, hence the maximum delay suffered from the ED $j\in V_{i}$. Each i∈D considers the delay associated to the direct link communication mode toward the SN S, and and the delivery delay resulted by its selection as relay node. Furthermore, both the potential delays are weighed by the degree of the considered node and by its coefficient of reputation. Hence, each ED i∈D evaluates both

(8) $$\delta_{i}^{\prime }=\frac{\delta_{S,i}}{deg^{d}\left(i\right)\rho_{i}+1};$$

and

(9) $$\delta_{i}^{\prime \prime }=\frac{\delta_{S,i}+{\bar{\delta }}_{i,j}}{deg^{d}\left(i\right)\rho_{i}+1};$$

### 4.3. Proposed Algorithm

In order to solve the problem formulated, we proposed a modified version of the Gale–Shapley algorithm (GSA) [33], which represents the state-of-the-art as regards the two-sided preferences matching problems [34,35,36]. More in depth, both the $S^{\prime }$ and $S^{\prime \prime }$ sets reside in S. More in depth, for the sake of simplicity, the S set has been split into $S^{\prime }$ and $S^{\prime \prime }$ that are a virtual representation of the two communication modes in which an ED can receive the packet *l*.

The algorithm reaches the suboptimal solution throughout an iterative bargaining process between $S^{\prime }$ or $S^{\prime \prime }$ and D. By defining $D^{\prime }$ and $D^{\prime \prime }$ as the set of proposals received by $S^{\prime }$ and $S^{\prime \prime }$ respectively, the algorithm steps are

1. each ED *i* constructs its preference accordingly to (8) and (9);
2. if ED *i* prefers $\delta_{i}^{\prime }$ to $\delta_{i}^{\prime \prime }$, i.e., the ED *i* prefers to receive the packet *l* through the direct link connection, *i* proposes itself to $S^{\prime }$, otherwise the proposal is sent to $S^{\prime \prime }$.
3. both $S^{\prime }$ and $S^{\prime \prime }$ select the minimum values $i^{\prime }$ and $i^{\prime \prime }$ within $D^{\prime }$ and $D^{\prime \prime }$, respectively.
4. Due to the fact that $i^{\prime }$ represents the first choice of $S^{\prime }$ among the received proposals, the SN S establishes a direct link towards $i^{\prime }$;
5. similarly, the SN S selects $i^{\prime \prime }$ as relay node in order to send the packet *l* to all the one hop neighbors of $i^{\prime \prime }$;
6. the $G_{d}$ topology is updated as follows:
  - the ED $i^{\prime }$ cuts its incident edges;
  - all the nodes belonging to $V_{i^{\prime \prime }}$ cut their incident edges, while preserving the edge connecting themselves with $i^{\prime \prime }$. In fact, the edges that connect $i^{\prime \prime }$ to its one hop neighbors are essential to reach all the neighbor EDs and to allow $i^{\prime \prime }$ to act as a relay.
7. $D=D\setminus V_{i^{\prime \prime }}\cup \left\{i^{\prime \prime },i^{\prime }\right\}$;
8. each ED i∈D updates its preference list;
9. repeat 1–9 until the set D is not empty.

Furthermore, it is dutiful to point out that $i^{\prime }$ or $i^{\prime \prime }$ may not exist, but at each round of the algorithm at least one of these terms exists, and if both $i^{\prime }$ and $i^{\prime \prime }$ exist, $i^{\prime }$ uses the direct link communication mode and $i^{\prime \prime }$ is selected as relay node.

### 4.4. Practical Considerations

In order to perform the time complexity analysis of the proposed approach, we focus on the worst case scenario. Under such an assumption, we suppose that each ED has the same preference as any other ED in D, and that the graph $G_{d}$ is completely disconnected. Within this context, it is straightforward to assume that $S^{\prime }$ receives exactly *n* proposals at the first step of the algorithm, n−1 at the second one, and so on. Consequently, given *n* proposals, the time complexity required to construct the preference list of $S^{\prime }$ over the received proposals is given by n·logn. Similarly, taking into account that at each iteration of the matching procedure the number of allocated ED is exactly one, due to the hypothesis of disconnection of the underlying graph, the time complexity of the proposed procedure is

(10) $$O\left(n\cdot 2\cdot \log2\right)+O\left(\sum_{w=0}^{n-1}\left(n-w\right)\cdot \log\left(n-w\right)\right),$$

where 2·log2 is the time complexity spent by each ED to build its preference list. It is important to note that each ED builds its preferences considering both the $S^{\prime }$ and $S^{\prime \prime }$ alternatives, and it justifies the cost equals to 2·log2 to build its preference list. In conclusion, the time complexity of the modified MT approach is in the order of

(11) O(n·logn).

## 5. Numerical Results

### 5.1. Simulation Setup

This section presents the system performance derived through computer simulations, applying the proposed matching game approach, a greedy algorithm based on the selection of the EDs at minimum delay (MDG), the random one (RA). Then, we have considered two well-known game theory based frameworks: the Kolkata paise restaurant game (KPRG) [37,38], the potential game approach (POT) [39,40], and the method proposed in [41], hereafter referred as REL.

The REL algorithm act as follows

- a threshold for the quality condition of the link towards each node is set. The threshold is expressed in terms of link delay;
- every link which satisfies the quality condition is selected as a relay link;
- the connection graph $G_{d}$ is updated in accordance with the degree of the nodes selected as relays;
- when any link has a quality greater than the fixed threshold, direct links are established until all the nodes are reached.

The considered MDG approach consists of the following steps:

1. the SN S establishes direct links with the disconnected EDs, according to the topology of $G_{d}$;
2. the SN S selects as a relay node the ED at minimum distance;
3. the topology of $G_{d}$ is updated;
4. repeat 1–4 until all the EDs are reached by one link.

Furthermore, the random algorithm has been adapted as follows:

1. the SN S selects the direct link communication mode with the disconnected EDs in $G_{d}$;
2. the SN S selects with uniform probability whether to establish direct link or to select a relay node;
3. the topology of $G_{d}$ is updated according to the previous decision;
4. repeat 1–4 until all the EDs are reached by one link.

The DLM acts by establishing a direct link between S and each ED. In order to apply the KPRG strategy to our problem scenario, we have modified the approach as follows:

- during each step, S establishes one direct link, as far as possible;
- during each step, $S^{\prime \prime }$ selects one relay, as far as possible;
- during each step, S establishes one direct link towards the most disconnected ED.
- during each step, $S^{\prime \prime }$ selects as a relay the ED with maximum degree.

Finally, we implemented the potential game approach already proposed in [39], but considering its sub-optimal version as in [40], in which the players involved in the game can deviate from the Nash equilibrium up to ϵ, with ϵ=0.01, in order to consider an alternative approach with an acceptable computational complexity.

### 5.2. Performance Analysis

The numerical results presented in this section arise from the simulation parameters detailed below. We consider a circular area network with a radius of 200 m, a D2D community located far away from the SN S, within a circular area with radius of 15 m. The transmission power between the SN S and one ED is set as $P_{S}=0.9$ W, while the D2D transmission power is $P_{i}=0.6$ W. The reputation coefficient $\rho_{i}$ assumes values within the interval (0,1). Finally, the path loss exponent α has been set to 2.5. The proximity graph $G_{d}$ is built establishing an edge between two EDs if their mutual distance is less than 10 m. The performance analysis is expressed in terms of delivery transmission delay, number of selected relays and, under the assumption of an equal power consumption for any ED acting as a relay, also in terms of overall power consumption.

**Figure 2:**
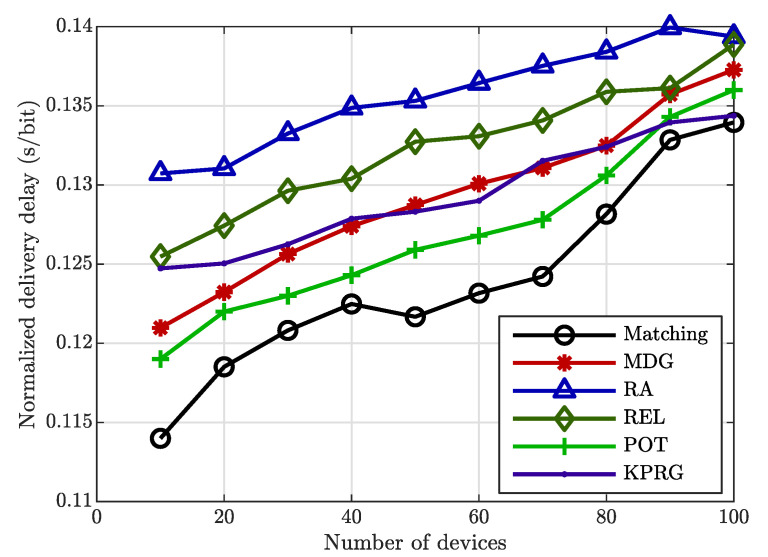
Delivery delay comparisons.

Figure 2 highlights the delivery delay normalized to the packet size achieved using the three different relay selection strategies. As can be seen, the proposed matching-based approach reaches lower values of delivery delay, ensuring a higher system responsiveness compared to the considered alternative approaches. As it is evident to note, by increasing the number of EDs, the delivery delay value decreases. This is due to the fact that the EDs are close to each other and the number of edges in $G_{d}$ grows with the size of the EDs set. Hence, with a greater ED density, the EDs are much closer to each other, and the delay is less than when the EDs density is low, i.e., when the EDs are more spread within the network area. Instead, in the same figure, the delay increases when the number of the EDs grows, since it is strictly related to the ρ coefficients. In fact, considering low values of EDs, the proposed approach is able to clearly select trusted (i.e., with high $\rho_{i}$ values) devices, avoiding the direct links. Instead, by increasing the density of the EDs in the selected area, our algorithm more often selects direct links, in order to guarantee a higher level of trustworthiness.

**Figure 3:**
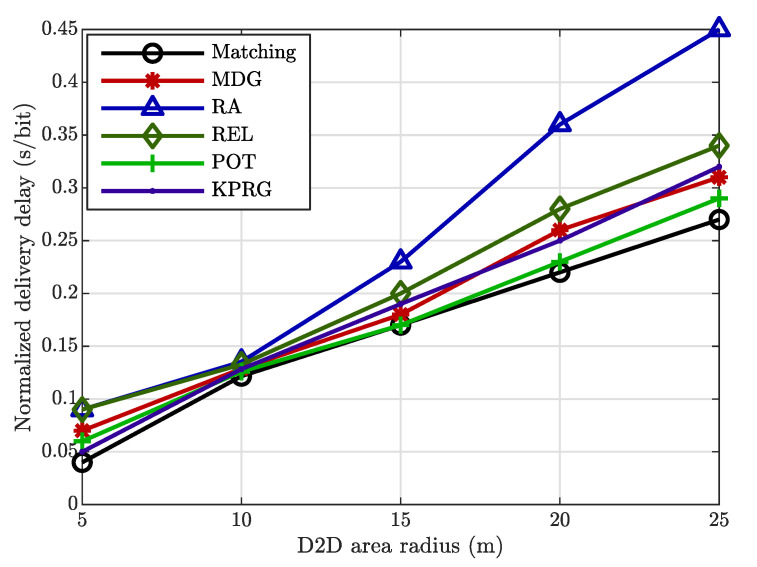
Normalized delivery delay comparisons for the case of 50 end devices (EDs).

Therefore, it is evident to note the remarkable advantages achieved by applying the relay selection in comparison to the alternatives. Furthermore, the previous result is confirmed by Figure 3, in which the number of EDs is fixed and equal to 50, and the D2D area radius changes within the set {5,10,15,20,25} m. The relay selection reaches better results when the radius has low values, hence when the D2D community is concentrated in a small area. Additionally, in this case the proposed algorithm has better performance for all the radius values.

**Figure 4:**
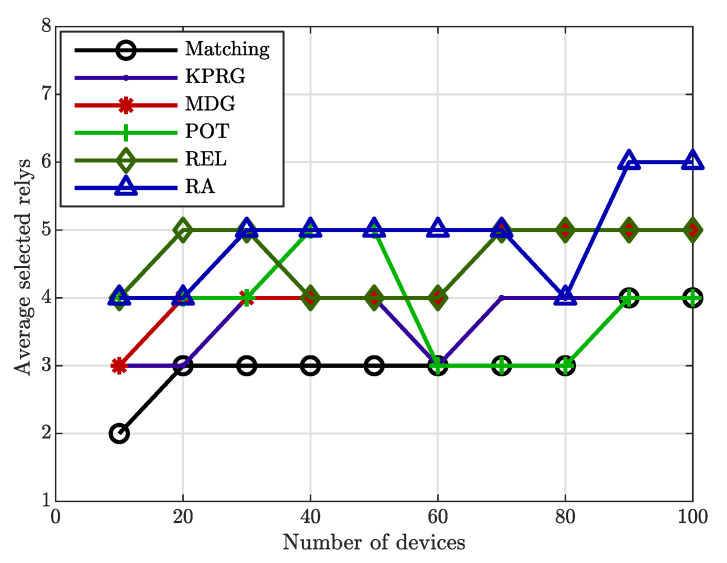
Number of selected relays.

**Figure 5:**
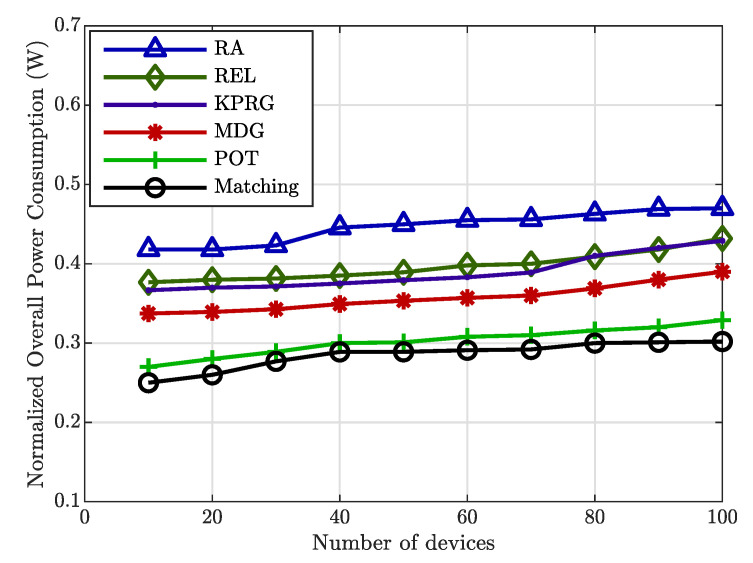
Normalized overall system power consumption comparison.

**Figure 6:**
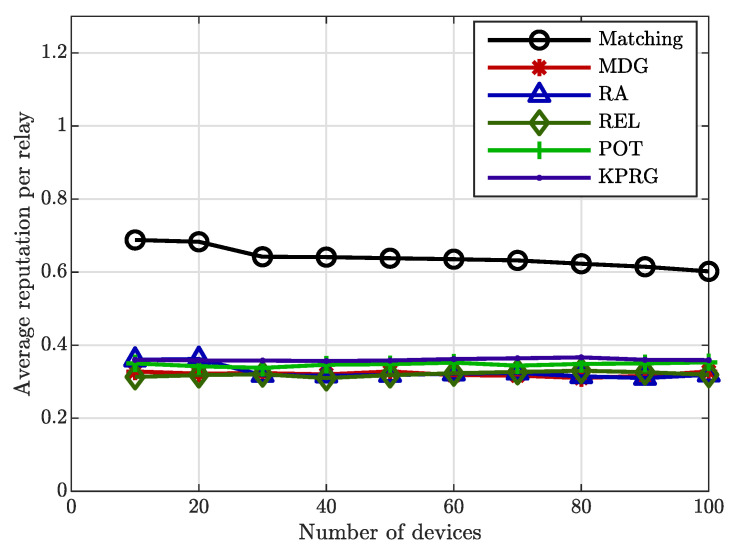
Average reputation level of relays.

Likewise, the validity of the proposed relay selection strategy is again evident in Figure 4, in which the number of the involved relays is lowered in comparison with the MDG and RA alternatives. Finally, Figure 5 illustrates the overall power consumption normalized to the individual relay power consumption (assumed equal for all the involved relays) as a function of the EDs number. This Figure highlights again a better behavior of the proposed algorithm in comparison with the considered alternatives. Figure 6 concludes our analysis by showing the impact of the reputation coefficient value on the relays selection. From this Figure, it is clearly evident that the proposed strategy favors the relays selection with high reputation levels.

## 6. Conclusions

This paper has focused attention on the multifaceted problem of the relay selection to contract the negative effects of channel propagation conditions in 5G networks on multicast communications with EDs at the network edge with the aim at lowering the overall energy consumption and the end-to-end delivery delay. In particular, the paper has proposed a suitable relay selection strategy, based on a matching game with one-sided preferences. It has been highlighted here that the proposed approach achieves better performance in comparison with the MDG, RA, REL, KPRG, and POT alternatives. Finally, we have also shown here that the proposed matching game approach provides good results even when the EDs in the D2D community are very close, hence, making the proposed algorithm suitable for dense/ultra dense networking currently considered as a promising technology for the forthcoming 5G network. In addition, future works include the deep investigation of the machine learning techniques applied to the relay selection schemes in the upcoming 5G networks, posing attention to the social relationships among the EDs and their human cognitive aspects, in order to adopt a user-centric perspective and a multi-disciplinary approach.

## Abbreviations

System variables:

Symbol	Description
S	source node
D	set of EDs
*n*	number of EDs in D
*l*	packet
*L*	packet size
$G_{d}$	proximity graph
$D_{d}$	vertex set of $G_{d}$
$E_{d}$	edges set of $G_{d}$
i,j	indices on set D
$\rho_{i}$	reputation coefficient
$deg^{d}\left(i\right)$	degree of vertex *i*
p(·)	probability function
$\delta_{S,i}$	transmission delay via direct link
$\delta_{S,j,i}$	transmission delay passing by ED *j*
$\Delta_{i}$	total delay transmission
R	number of selected relays

## References

[1] Asadi A., Wang Q., Mancuso V. (2014). A Survey on Device-to-Device Communication in Cellular Networks. IEEE Commun. Surv. Tutor..

[2] (2020). Release 12 3gpp. https://www.3gpp.org/specifications/releases/68-release-12.

[3] (2020). Specification Details 3gpp. https://portal.3gpp.org/desktopmodules/Specifications/SpecificationDetails.aspx?specificationId=3389.

[4] Bello O., Zeadally S. (2016). Intelligent Device-to-Device Communication in the Internet of Things. IEEE Syst. J..

[5] Tehrani M.N., Uysal M., Yanikomeroglu H. (2014). Device-to-device communication in 5G cellular networks: Challenges, solutions, and future directions. IEEE Commun. Mag..

[6] Höyhtyä M., Apilo O., Lasanen M. (2018). Review of Latest Advances in 3GPP Standardization: D2D Communication in 5G Systems and Its Energy Consumption Models. Future Internet.

[7] Chiti F., Giacomo D.D., Fantacci R., Pierucci L. Interference aware approach for D2D communications. Proceedings of the 2016 IEEE International Conference on Communications (ICC).

[8] Biswas S., Vuppala S., Xue J., Ratnarajah T. (2016). On the Performance of Relay Aided Millimeter Wave Networks. IEEE J. Sel. Top. Signal Process..

[9] Wu S., Atat R., Mastronarde N., Liu L. (2018). Improving the Coverage and Spectral Efficiency of Millimeter-Wave Cellular Networks Using Device-to-Device Relays. IEEE Trans. Commun..

[10] Wei L., Cao Z., Zhu H. MobiGame: A User-Centric Reputation Based Incentive Protocol for Delay/Disruption Tolerant Networks. Proceedings of the 2011 IEEE Global Telecommunications Conference-GLOBECOM 2011.

[11] Desogus C., Anedda M., Murroni M., Muntean G. (2019). A Traffic Type-Based Differentiated Reputation Algorithm for Radio Resource Allocation During Multi-Service Content Delivery in 5G Heterogeneous Scenarios. IEEE Access.

[12] Kar U.N., Sanyal D.K. (2020). A Critical Review of 3GPP Standardization of Device-to-Device Communication in Cellular Networks. SN Comput. Sci..

[13] Zlatanov N., Jamali V., Schober R. (2015). Achievable Rates for the Fading Half-Duplex Single Relay Selection Network Using Buffer-Aided Relaying. IEEE Trans. Wirel. Commun..

[14] Simoni R., Jamali V., Zlatanov N., Schober R., Pierucci L., Fantacci R. (2016). Buffer-Aided Diamond Relay Network With Block Fading and Inter-Relay Interference. IEEE Trans. Wirel. Commun..

[15] Krikidis I., Charalambous T., Thompson J.S. (2012). Buffer-Aided Relay Selection for Cooperative Diversity Systems without Delay Constraints. IEEE Trans. Wirel. Commun..

[16] Sarker D.K., Sarkar M.Z.I., Anower M.S. Enhancing multicast capacity using opportunistic relaying. Proceedings of the 2016 2nd International Conference on Electrical, Computer Telecommunication Engineering (ICECTE).

[17] Wang Y., Wang W., Chen L., Zhang Z. Lexicographic Relay Selection and Channel Allocation for Multichannel Cooperative Multicast. Proceedings of the 2017 IEEE Wireless Communications and Networking Conference (WCNC).

[18] Mishra P.K., Pandey S., Biswash S.K. (2016). A Device-Centric Scheme for Relay Selection in a Dynamic Network Scenario for 5G Communication. IEEE Access.

[19] Shama N., Saxena N., Roy A. (2020). Incentive and Penalty Mechanism For Power Allocation in Cooperative D2D-Cellular Transmissions. Electronics.

[20] Zhao Y., Li Y., Cao Y., Jiang T., Ge N. (2015). Social-Aware Resource Allocation for Device-to-Device Communications Underlaying Cellular Networks. IEEE Trans. Wirel. Commun..

[21] Chiti F., Fantacci R., Pierucci L. (2017). Social-Aware Relay Selection for Cooperative Multicast Device-to-Device Communications. Future Internet.

[22] Zhang H., Wang Z., Du Q. (2018). Social-Aware D2D Relay Networks for Stability Enhancement: An Optimal Stopping Approach. IEEE Trans. Veh. Technol..

[23] Huang S., Wei Z., Yuan X., Feng Z., Zhang P. (2017). Performance Characterization of Machine-to-Machine Networks With Energy Harvesting and Social-Aware Relays. IEEE Access.

[24] Khwakhali U.S., Gordon S., Suksompong P. Social-aware relay selection for device to device communications in cooperative cellular networks. Proceedings of the 2017 International Electrical Engineering Congress (iEECON).

[25] Xu C., Gao C., Zhou Z., Chang Z., Jia Y. (2017). Social Network-Based Content Delivery in Device-to-Device Underlay Cellular Networks Using Matching Theory. IEEE Access.

[26] Xu L., Junhao F., Biyao H., Zhou Z., Mumtaz S., Rodriguez J. (2017). Joint Relay Selection and Resource Allocation for Energy-Efficient D2D Cooperative Communications Using Matching Theory. Appl. Sci..

[27] Biró P., Irving R.W., Manlove D.F., Calamoneri T., Diaz J. (2010). Popular Matchings in the Marriage and Roommates Problems. Algorithms and Complexity.

[28] Sawyer N., Smith D.B. (2019). Flexible Resource Allocation in Device-to-Device Communications Using Stackelberg Game Theory. IEEE Trans. Commun..

[29] Bayat S., Li Y., Song L., Han Z. (2016). Matching Theory: Applications in wireless communications. IEEE Signal Process. Mag..

[30] Han Z., Niyato D., Saad W., Basar T., Hjørungnes A. (2017). Matching Theory for Wireless Networks.

[31] Gu Y., Saad W., Bennis M., Debbah M., Han Z. (2015). Matching theory for future wireless networks: Fundamentals and applications. IEEE Commun. Mag..

[32] Manlove D. (2013). Algorithmics Of Matching Under Preferences.

[33] Gale D., Shapley L.S. (1962). College Admissions and the Stability of Marriage. Am. Math. Mon..

[34] Roth A.E. (2008). Deferred acceptance algorithms: History, theory, practice, and open questions. Int. J. Game Theory.

[35] Roth A.E., Sotomayor M. (1990). Two-Sided Matching: A Study in Game-Theoretic Modeling and Analysis.

[36] El-Atta A.H.A., Moussa M.I. Student Project Allocation with Preference Lists over (Student, Project) Pairs. Proceedings of the 2009 Second International Conference on Computer and Electrical Engineering.

[37] Chakrabarti A.S., Chakrabarti B.K., Chatterjee A., Mitra M. (2009). The Kolkata Paise Restaurant problem and resource utilization. Phys. A Stat. Mech. Appl..

[38] Park T., Saad W. Kolkata paise restaurant game for resource allocation in the Internet of Things. Proceedings of the 2017 51st Asilomar Conference on Signals, Systems, and Computers.

[39] Zhong W., Chen G., Jin S., Wong K. (2014). Relay Selection and Discrete Power Control for Cognitive Relay Networks via Potential Game. IEEE Trans. Signal Process..

[40] Shah-Mansouri H., Wong V.W.S. (2018). Hierarchical Fog-Cloud Computing for IoT Systems: A Computation Offloading Game. IEEE Internet Things J..

[41] Tian Z., Gong Y., Chen G., Chambers J.A. (2017). Buffer-Aided Relay Selection With Reduced Packet Delay in Cooperative Networks. IEEE Trans. Veh. Technol..

